# Risk factors of vertebral re-fracture after PVP or PKP for osteoporotic vertebral compression fractures, especially in Eastern Asia: a systematic review and meta-analysis

**DOI:** 10.1186/s13018-022-03038-z

**Published:** 2022-03-12

**Authors:** Chuanqiang Dai, Gang Liang, Youshu Zhang, Yao Dong, Xiaodan Zhou

**Affiliations:** 1 Department of Orthopaedics, The First People’s Hospital of Ziyang, Ziyang, Sichuan China; 2 Department of Orthopedics, Fuzong Clinical Medical College of Fujian Medical University, Fuzhou, 350025 Fujian China

**Keywords:** Percutaneous vertebroplasty, Percutaneous kyphoplasty, Osteoporotic vertebral compression fracture, Refracture, Risk factors, Meta-analysis

## Abstract

**Objective:**

Percutaneous vertebroplasty (PVP) and kyphoplasty (PKP) have been widely used to treat osteoporotic vertebral compression fractures (OVCF), but the risk of vertebral re-fracture after PVP/PKP remains controversial. This study aims to investigate the incidence and risk factors of vertebral re-fracture after PVP/PKP.

**Methods:**

Relevant literatures published up to November 2021 were collected from PubMed, Embase and Web of Science. A meta-analysis was performed to extract data associated with risk factors of SVCF following the PRISMA guidelines. Also, pooled odds ratio (OR) or weighted mean difference (WMD) with 95% confidence interval (CI) was calculated.

**Results:**

A total of 23 studies, encompassing 9372 patients with OVCF, met the inclusion criteria. 1255 patients (13.39%) suffered re-fracture after PVP/PKP surgery. A total of 22 studies were from Eastern Asia and only 1 study was from Europe. Female sex (OR = 1.34, 95%CI 1.09–1.64, *P* = 0.006), older age (WMD = 2.04, 95%CI 0.84–3.24, *P* = 0.001), lower bone mineral density (BMD, WMD = − 0.38, 95%CI − 0.49–0.26, *P* < 0.001) and bone cement leakages (OR = 2.05, 95% CI 1.40–3.00, *P* < 0.001) increased the risk of SVCF. The results of subgroup analysis showed the occurrence of re-fracture was significantly associated with gender (*P* = 0.002), age (*P* = 0.001) and BMD (*P* < 0.001) in Eastern Asia. Compared with the unfractured group, anterior-to-posterior vertebral body height ratio (AP ratio, WMD = 0.06, 95%CI 0.00–0.12, *P* = 0.037) and visual analog scale score (VAS, WMD = 0.62, 95%CI 0.09–1.15, *P* = 0.022) were higher in the refracture group, and kyphotic angle correction ratio (Cobb ratio, WMD = − 0.72, 95%CI − 1.26–0.18, *P* = 0.008) was smaller in Eastern Asia. In addition, anti-osteoporosis treatment (OR = 0.40, 95% CI 0.27–0.60, *P* < 0.001) could be a protective factor.

**Conclusion:**

The main factors associated with re-fracture after PVP/PKP are sex, age, bone mineral density, AP ratio, Cobb ratio, VAS score, bone cement leakage and anti-osteoporosis treatment, especially in Eastern Asia.

## Introduction

Osteoporosis has been identified by the World Health Organization as one of the top 10 most serious diseases worldwide [1, 2]. As the global population continues to age, the incidence of osteoporotic vertebral compression fracture (OVCF) is increasing year by year, affecting more than 1.4 million people worldwide each year, and has become a non-negligible problem of the elderly [3, 4]. Also, OVCF can cause chronic back pain, limited mobility, impaired physical function, reduced quality of life and increased mortality in older patients [5, 6].

Percutaneous vertebroplasty (PVP) and percutaneous kyphoplasty (PKP) are minimally invasive techniques widely used in OVCF, due to their promising application prospects and encouraging clinical outcomes [7–11]. However, some studies have suggested that secondary vertebral compression fractures (SVCF) are associated with PVP/PKP surgery for patients with OVCF [12–15]. It has been shown that prolonged menopause and low bone mineral density (BMD) may increase the risk of re-fracture after PVP [16]. Body mass index (BMI), preoperative multisegmental vertebral compression fractures, and dispersion degree of bone cement have also been reported to be related to re-fractures after PVP or PKP [17–19]. Overall, the relationship between the occurrence of vertebral fractures and PVP/PKP surgery remains controversial due to the varying results of clinical trials.

Appropriate management and risk avoidance could reduce the incidence of SVCF. Therefore, we focused on the incidence of re-fracture and the risk factors for re-fracture in OVCF patients after PVP/PKP. The collected studies were comprehensively evaluated through meta-analysis. This meta-analysis may serve as reliable evidence for further prevention of re-fracture after PVP/PKP. The pooled results can provide relevant data for the subsequent development and validation of a prediction tool for re-fracture after PVP/PKP.

## Method

### Information sources and literature search

This systematic review and meta-analysis were in accordance with the Preferred Reporting Items for Systematic Reviews (PRISMA) guidelines and based on the Meta-analysis of Observational Studies in Epidemiology (MOOSE) statement [21, 22]. A systematic literature search of the PubMed, Embase, Web of Science was conducted by two researchers for relevant studies published up to November 2021. To collect studies on the incidence and risk factors of refracture after PVP/ PKP, we used the following search terms in the title, abstract or list of medical subject headings: "Osteoporotic vertebral compression fractures", "OVCRF" OR "Osteoporotic spinal fractures" AND "percutaneous vertebroplasty", "PVP" or "percutaneous balloon kyphoplasty", "percutaneous kyphoplasty", "PKP" OR "refractures", "re-fracture", "new vertebral compression fractures", "NVCF", "secondary vertebral compression fractures" OR "SVCF". Obviously irrelevant studies were excluded by browsing the titles and abstracts, and then the full text of the remaining literature was read to determine eligible publications.

### Study selection

The included literature must meet the following criteria: (1) The subjects were OVCF patients who received PVP or PKP treatment; (2) The study was a randomized controlled study or observational study, including cohort studies and case–control studies; (3) The study reported the incidence of refractures or assessed the risk factors associated with refractures; (4) The study involved more than 50 participants; (5) The study published in English only. Case reports, systematic reviews, letters, comments, and conference reports were excluded. If the study subjects were other animals, treatments other than PVP and PKP were used, or clinical data were not available, they would be excluded. If multiple studies reported the same data set, we included studies with longer follow-up and more detailed reporting of risk factors.

### Quality assessment and data extraction

The Newcastle–Ottawa scale (NOS) was used to assess the quality of the included articles [23]. A study can be given a maximum of 1 point for each item within the Selection and Exposure categories, and a maximum of 2 points for Comparability. The highest NOS score was 9 points, and the lowest was 0 points. The studies with a NOS score ≥ 6 were considered high quality and included in this meta-analysis.

Two researchers extracted relevant data from qualified studies using standardized forms. The first was the general characteristics of the eligible study, including first author, study region, year of publication, sample size, treatment method (PVP or PKP), average age, gender, and follow-up time. The primary outcome was the incidence and risk factors of refracture. Refractures included adjacent vertebral fractures and distal vertebral fractures. If there were more than three studies reporting the same indicator, the indicator would be used as a candidate risk factor for meta-analysis. The risk factors assessed in this meta-analysis included gender, age, BMI, BMD, number of vertebral compression fractures (VCF), bone cement volume, bone cement leakage, anterior-to-posterior vertebral body height ratio (AP ratio), kyphotic angle correction ratio (Cobb ratio), visual analog scale (VAS) score and postoperative anti-osteoporosis treatment. If the corresponding data could not be extracted directly from the study, re-analysis was required. Also, if there was any disagreement between the 2 researchers, it was resolved through discussion or by a third researcher.

### Statistical analysis

The extracted data was imported into STATA 16.0 software for statistical analysis. We used a random-effects model to aggregate odds ratio (OR) or weighted mean difference (WMD) with 95% confidence interval (CI). Dichotomous data were analyzed using OR, and continuous data were evaluated using the WMD. The aggregated results were presented in a forest plot. The heterogeneity between studies was evaluated by *I*^2^ statistics and Q test [24]. *I*^2^ > 50% and *P* < 0.1 indicated significant heterogeneity [25]. Subgroup analyses were performed according to whether the included studies were from Eastern Asia. When the number of studies involved was ≥ 10, the Begg’s test was used to assess publication bias. *P* value was calculated by two-tailed test, and *P* < 0.05 was considered statistically significant.

## Results

### Search result

**Fig. 1:**
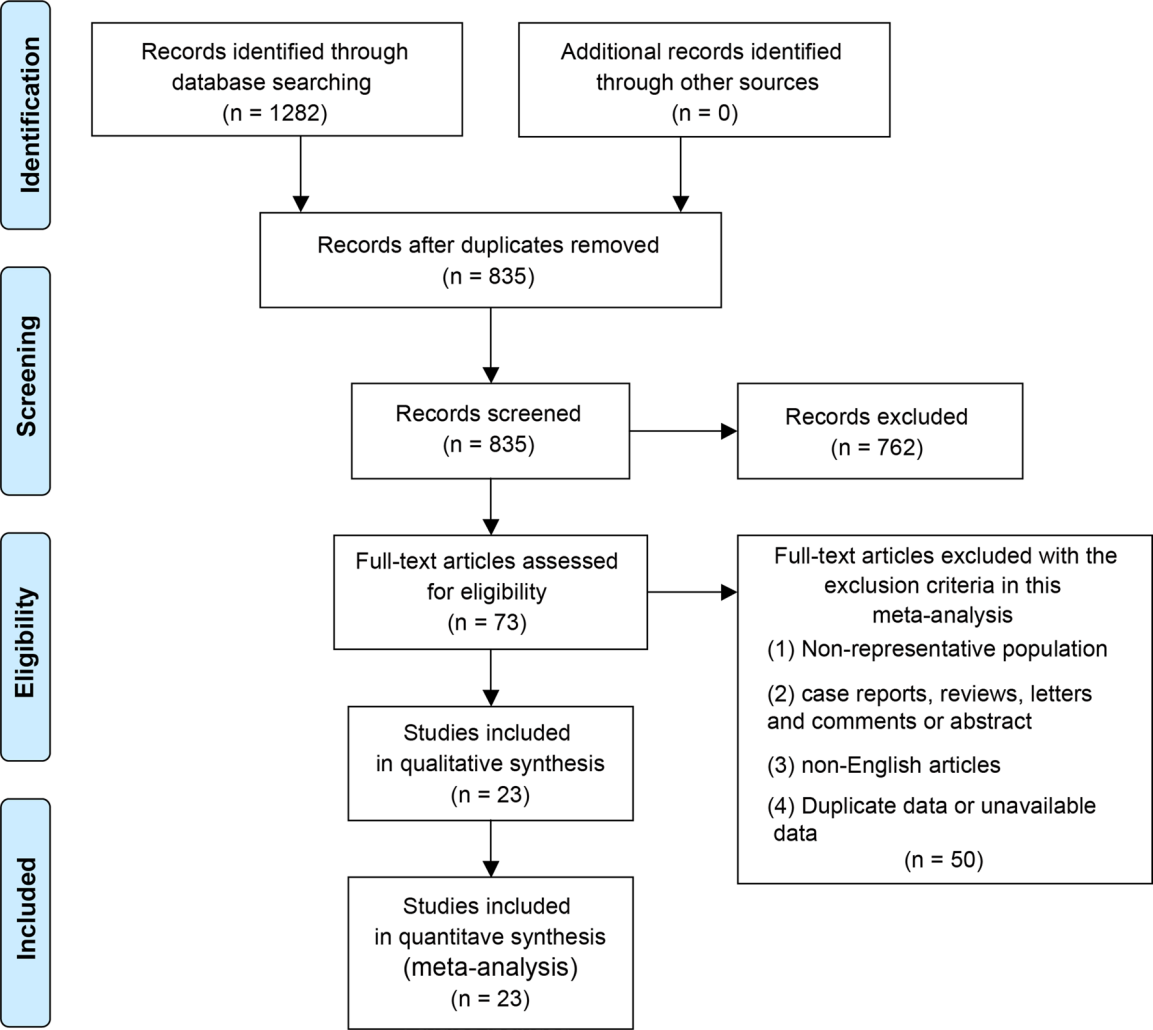
Flowchart of search strategy for included studies

According to the retrieval strategy, we first collected 1282 publications. After removing duplicates, the remaining 835 studies were screened for titles and abstracts. Subsequently, 73 studies were eligible for full text review. Among them, 50 studies were further excluded because they did not meet the inclusion criteria. Finally, a total of 23 studies were included in this systematic review and meta-analysis [5, 16, 18, 20, 26–44]. The process and results of literature screening were shown in Fig. 1.

### Literature characteristics

The basic characteristics of the included studies were shown in Table 1. A total of 9372 patients were included in 23 studies, of which 1255 patients suffered re-fractures after surgery, with an incidence of 13.39%. These studies were carried out in 5 regions, including 7 studies in South Korea [18, 32, 38, 39, 41, 42, 44], 13 studies in China [5, 16, 20, 26–31, 33–36], 1 study in Taiwan [40], 1 study in Japan [37], and 1 study in Belgium [43]. A total of 22 studies were from Eastern Asia and only 1 study was from Europe. The included studies were published between 2006 and 2021. The average follow-up time was 21.4 months. The average age of the enrolled patients was 72.0 years. Four studies included only female patients [16, 29, 37, 42]. The average NOS score of the included studies was 7.78, 4 studies scored 9 [26, 28, 33, 44], 11 studies scored 8 [20, 27, 29–32, 36, 38–41], 7 studies scored 7 [5, 18, 34, 35, 37, 42, 43], 1 study score was 6 [16], all of which were high-quality studies.

**Table 1:** Characteristics of the eligible study in this meta-analysis

Author	Year	Region	Study design	Operation style	All patients	Patients with re-fracture	Age (years)	Follow-up	Outcome	NOS score
Bae et al. [18]	2017	Korea	Retrospective study	PVP	265	34	71.9	36	Cement leakage, cobb ratio	7
Chen et al. [40]	2011	Taiwan	Retrospective study	PVP	1800	10	77.71	24	Gender, age, cement volume, cobb ratio	8
Chen et al. [33]	2019	China	Retrospective study	PVP/PKP	650	102	73	24	Gender, cement leakage, anti-osteoporosis	9
Chen et al. [30]	2020	China	Retrospective study	PKP	102	16	71.1	12	Gender, age, cement volume, VCF, VAS, cobb ratio	8
Fang et al. [28]	2021	China	Retrospective study	PKP	228	24	69.7	28.8	Gender, BMI, age, anti-osteoporosis	8
Heo et al. [41]	2009	Korea	Retrospective study	PVP	343	11	68.1	4	Gender, BMD, age, cement volume, AP ratio	8
Hu et al. [5]	2019	China	Retrospective study	PVP	112	28	73.8	5.9	Gender, BMD, age	7
Lee et al. [44]	2006	Korea	Retrospective study	PVP	244	38	66.4	52.5	Gender, age, AP ratio	9
Lee et al. [38]	2015	Korea	Retrospective study	PVP	198	34	76.6	48.2	Gender	8
Lee et al. [32]	2019	Korea	Retrospective study	PVP	323	91	74.68	4.4	BMI, BMD, age, cement volume, cement leakage	8
Li et al. [16]	2017	China	Retrospective study	PVP	390	68	69.9	18	Age, VCF, anti-osteoporosis	6
Li et al. [34]	2018	China	Retrospective study	PVP/PKP	230	30	71.83	12	Gender, BMI, BMD, cement volume, cobb ratio	7
Lin et al. [35]	2017	China	Retrospective study	PKP	495	110	72	12	Gender, BMD, cement volume, cement leakage, cobb ratio, VAS	7
Lu et al. [29]	2020	China	Retrospective study	PVP	101	13	65.54	24	BMI, BMD, cement volume, cement leakage, cobb ratio	8
Moon et al. [42]	2007	Korea	Retrospective study	PKP	111	20	74.9	15.2	BMI, BMD, age, VCF	7
Ning et al. [27]	2021	China	Retrospective study	PKP	921	111	72.06	42.63	gender, BMI, BMD, age, anti-osteoporosis	9
Rho et al. [39]	2012	Korea	Retrospective study	PVP/PKP	147	27	70.03	35.5	Gender, BMD, age, cement volume, AP ratio, cement leakage	8
Takahara et al. [37]	2016	Japan	Retrospective study	PVP	61	14	78.9	12	BMI, age, VAS	7
Voormolen et al. [43]	2006	Belgium	Retrospective study	PVP	66	16	70	12	Gender, BMD, age, cement volume	7
Yang et al. [36]	2016	China	Retrospective study	PKP	139	21	75.9	19.56	Gender, BMI, BMD, age, cement volume, VCF, cement leakage, VAS	8
Yu et al. [31]	2019	China	Retrospective study	PVP	152	42	70.22	27.47	Gender, BMD, age, cement volume, cement leakage, VAS	8
Zhang et al. [26]	2021	China	Retrospective Study	PVP	2202	362	66.4	14.7	Gender, age, cement volume, VCF, cement leakage, anti-osteoporosis	8
Zhao et al. [25]	2021	China	Retrospective Study	PKP	92	33	74.9	8.2	Gender, BMI, age	9

AP ratio, anterior–posterior ratio; BMD, bone mineral density; BMI, Body Mass Index; IVC, intravertebral vacuum; VAS, visual analogue scale; VCF, vertebral compression fractures

### Meta-analysis of risk factors contributing to SVCF

We conducted a meta-analysis of 11 reported risk factors: gender, age, BMI, BMD, number of fractured vertebrae, bone cement volume, bone cement leakage, AP ratio, Cobb ratio, VAS and postoperative anti-osteoporosis treatment.

**Fig. 2:**
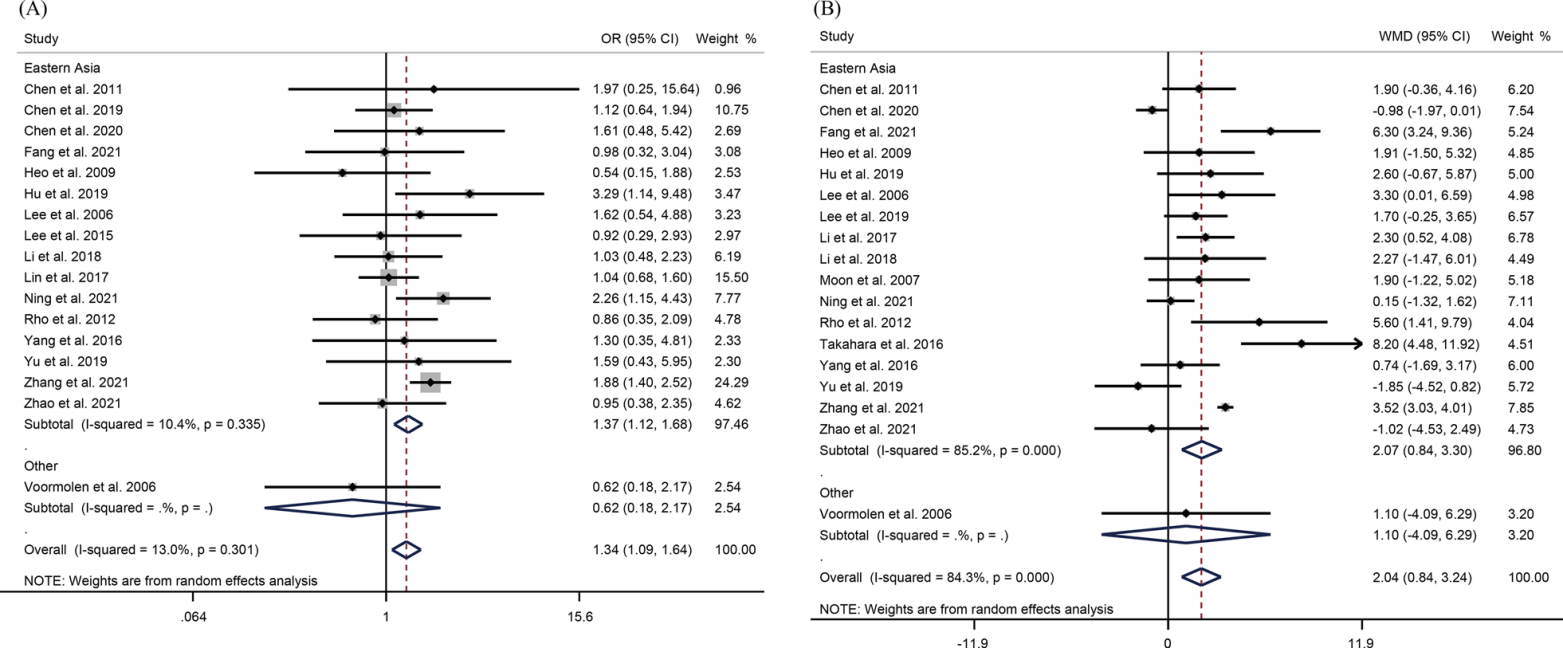
Forest plot of **A** gender and **B** age as risk factors for re-fracture after PVP/ PKP in OVCF patients in a random-effect model meta-analysis. OVCF, osteoporotic vertebral compression fracture. PVP, percutaneous vertebroplasty. PKP, percutaneous kyphoplasty

**Fig. 3:**
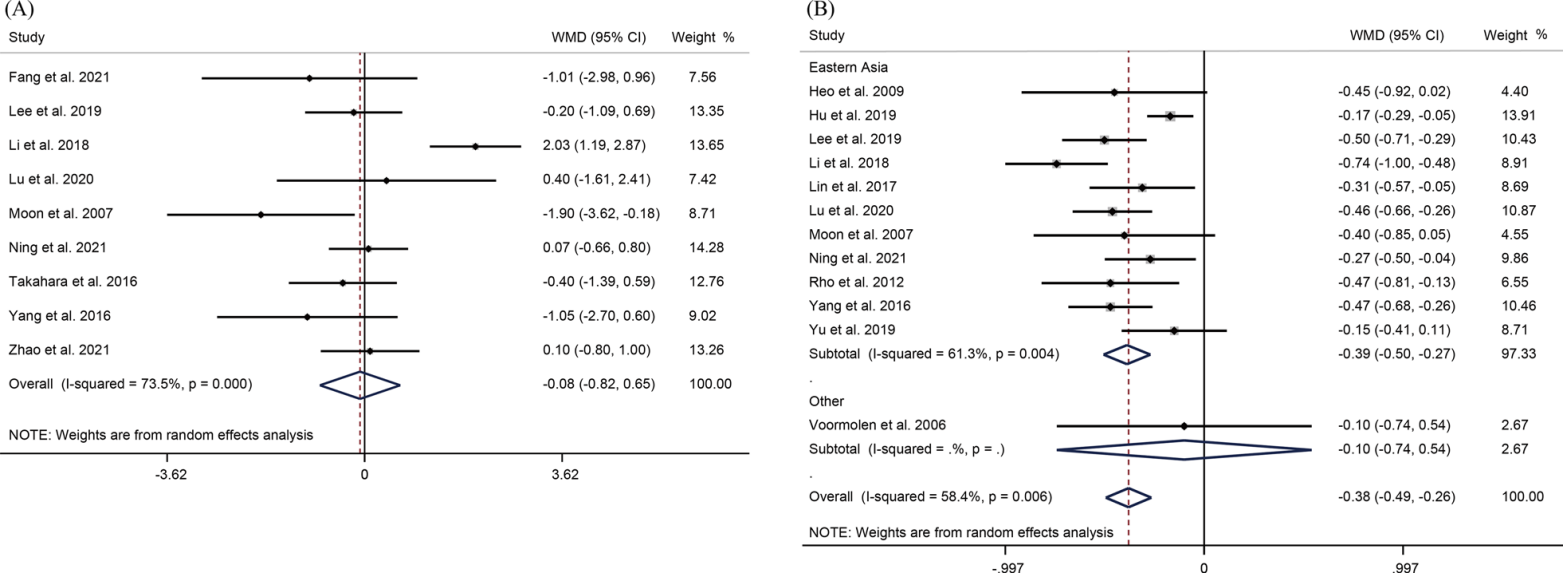
Forest plot of **A** BMI and **B** BMD as risk factors for re-fracture after PVP/ PKP in OVCF patients in a random-effect model meta-analysis. BMI, body mass index. BMD, bone density. OVCF, osteoporotic vertebral compression fracture. PVP, percutaneous vertebroplasty. PKP, percutaneous kyphoplasty

Seventeen studies reported the gender of the patient. The pooled OR was 1.34 (95%CI 1.09–1.64, *P* = 0.006), suggesting that female was a risk factor for re-fracture after PVP/PKP (Fig. 2A). According to subgroup analyses stratified by regions, the occurrence of re-fracture after PVP/PKP was significantly associated with patient gender in Eastern Asia (OR = 1.37, 95%CI 1.12–1.68, *P* = 0.002) without significant heterogeneity (*I*^2^ = 10.4%, *P* = 0.34). Eighteen studies mentioned the age of patients. The results showed that patients with SVCF were older than those without fractures (WMD = 2.04, 95% CI 0.84–3.24, *P* = 0.001) (Fig. 2B). In Eastern Asia, elderly patients had a significantly increased risk of re-fracture. Also, there was no significant correlation between the occurrence of SVCF and BMI of patients (WMD = − 0.08, 95% CI − 0.82–0.65, *P* = 0.82), and with significant heterogeneity (*I*^2^ = 73.5%, *P* < 0.001) (Fig. 3A). A total of 12 studies explored whether BMD could be treated as a risk factor. The WMD was − 0.38 (95%CI − 0.49 to 0.26, *P* < 0.001), indicating that low BMD was a risk factor for SVCF after PVP/PKP in OVCF patients (Fig. 3B). In term of region, lower BMD was significantly related to the occurrence of SVCF in Eastern Asia (WMD = − 0.39, 95% CI − 0.50 to 0.27, *P* < 0.001) with significant heterogeneity (*I*^2^ = 61.3%, *P* = 0.004).

**Fig. 4:**
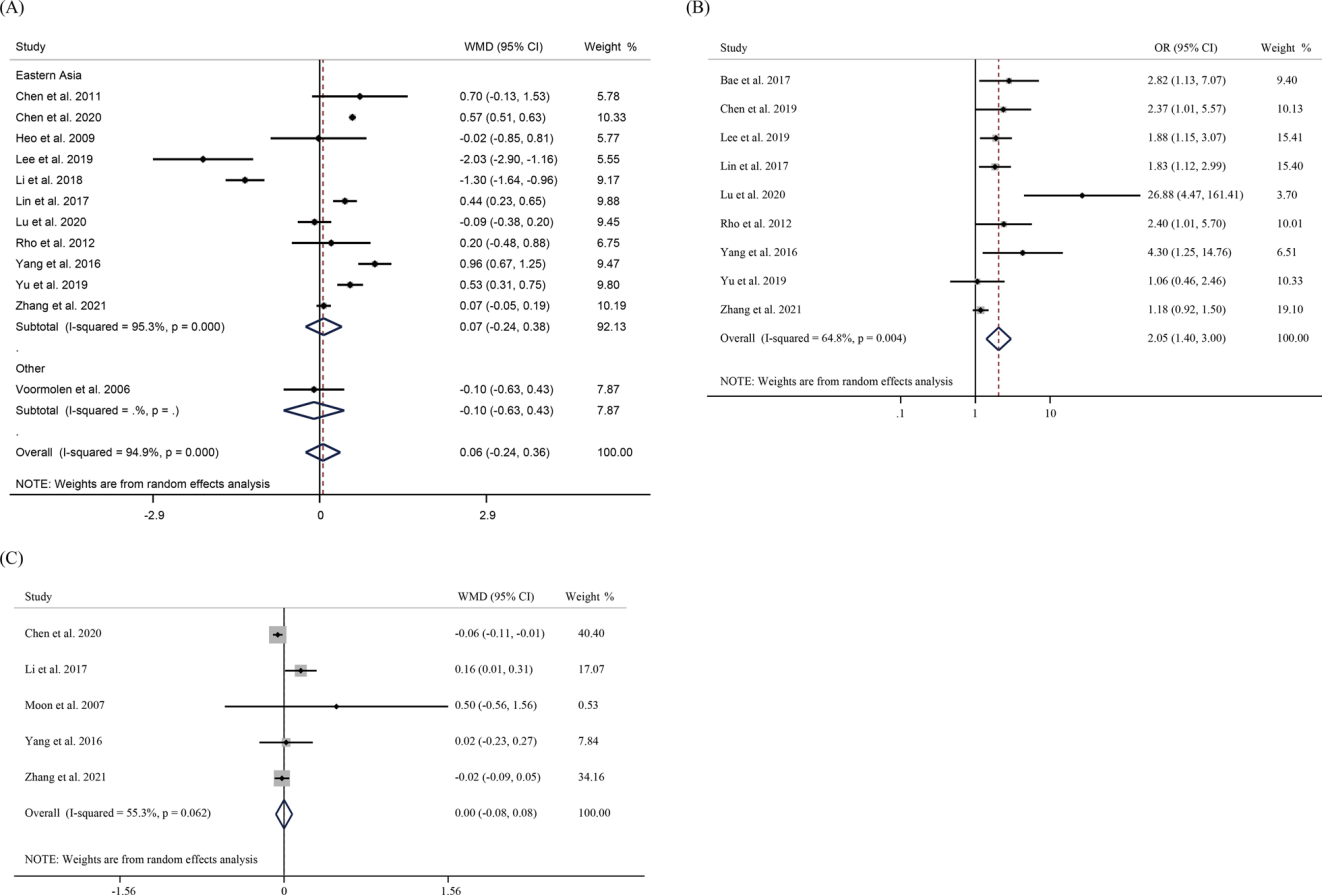
Forest plot of **A** bone cement volume, **B** bone cement leakage and **C** number of VCF as risk factors for re-fracture after PVP/ PKP in OVCF patients in a random-effect model meta-analysis. VCF, vertebral compression fracture. OVCF, osteoporotic vertebral compression fracture. PVP, percutaneous vertebroplasty. PKP, percutaneous kyphoplasty

Twelve studies involved the volume of bone cement injected into the patients. The random-effects meta-analysis suggested that bone cement volume was not a risk factor for SVCF (WMD = 0.06, 95% CI − 0.24–0.36, *P* = 0.711) (Fig. 4A). The results of subgroup analysis showed no significant correlation between re-fracture and cement volume after PVP/PKP, whether in Eastern Asia (WMD = 0.007, 95% CI − 0.24 to 0.38, *P* = 0.66) or other regions (WMD = − 0.10, 95% CI − 0.63 to 0.43, *P* = 0.71). In terms of bone cement leakage, the pooled OR was 2.05 (95%CI 1.40–3.00, *P* < 0.001), indicating that patients with bone cement leakage were more likely to develop SVCF after PVP/PKP (Fig. 4B). Five studies involving 2944 patients reported the number of VCF. The pooled WMD was 0.00 (95%CI − 0.08 to 0.08, *P* = 0.991), suggesting no significant correlation between the number of fractured vertebrae and postoperative refracture (Fig. 4C).

**Fig. 5:**
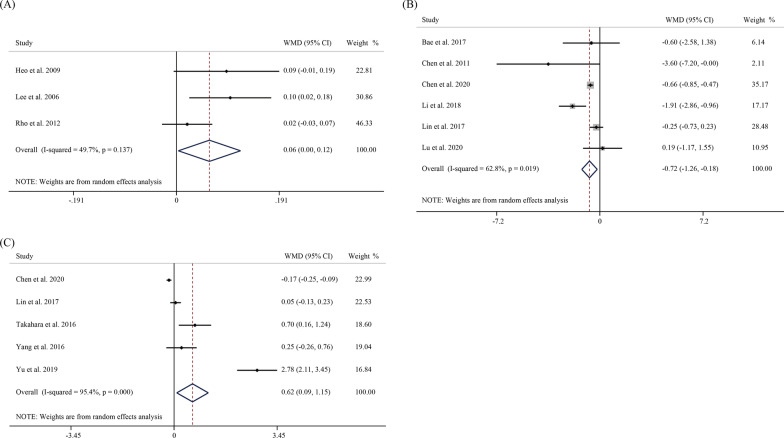
Forest plot of **A** A:P ratio, **B** Cobb ratio and **C** VAS as risk factors for re-fracture after PVP/ PKP in OVCF patients in a random-effect model meta-analysis. AP ratio, anterior–posterior ratio. VAS, visual analog scale. OVCF, osteoporotic vertebral compression fracture. PVP, percutaneous vertebroplasty. PKP, percutaneous kyphoplasty

AP ratio was reported in three studies involving 734 patents from Eastern Asia. The aggregated results showed that patients with a high AP ratio were more likely to develop SVCF (WMD = 0.06, 95% CI 0.00–0.12, *P* = 0.037) (Fig. 5A). Moreover, a total of 6 studies explored the Cobb ratio. The pooled WMD indicated that Cobb ratio could be a risk factor for SVCF (WMD = 0.06, 95% CI 0.00–0.12, *P* = 0.037) (Fig. 5B). Five studies involving 843 patients explored the postoperative VAS score as a risk factor for postoperative re-fracture, with WMD of 0.62 (95% CI 0.09–1.15, *P* = 0.022) (Fig. 5C).

**Fig. 6:**
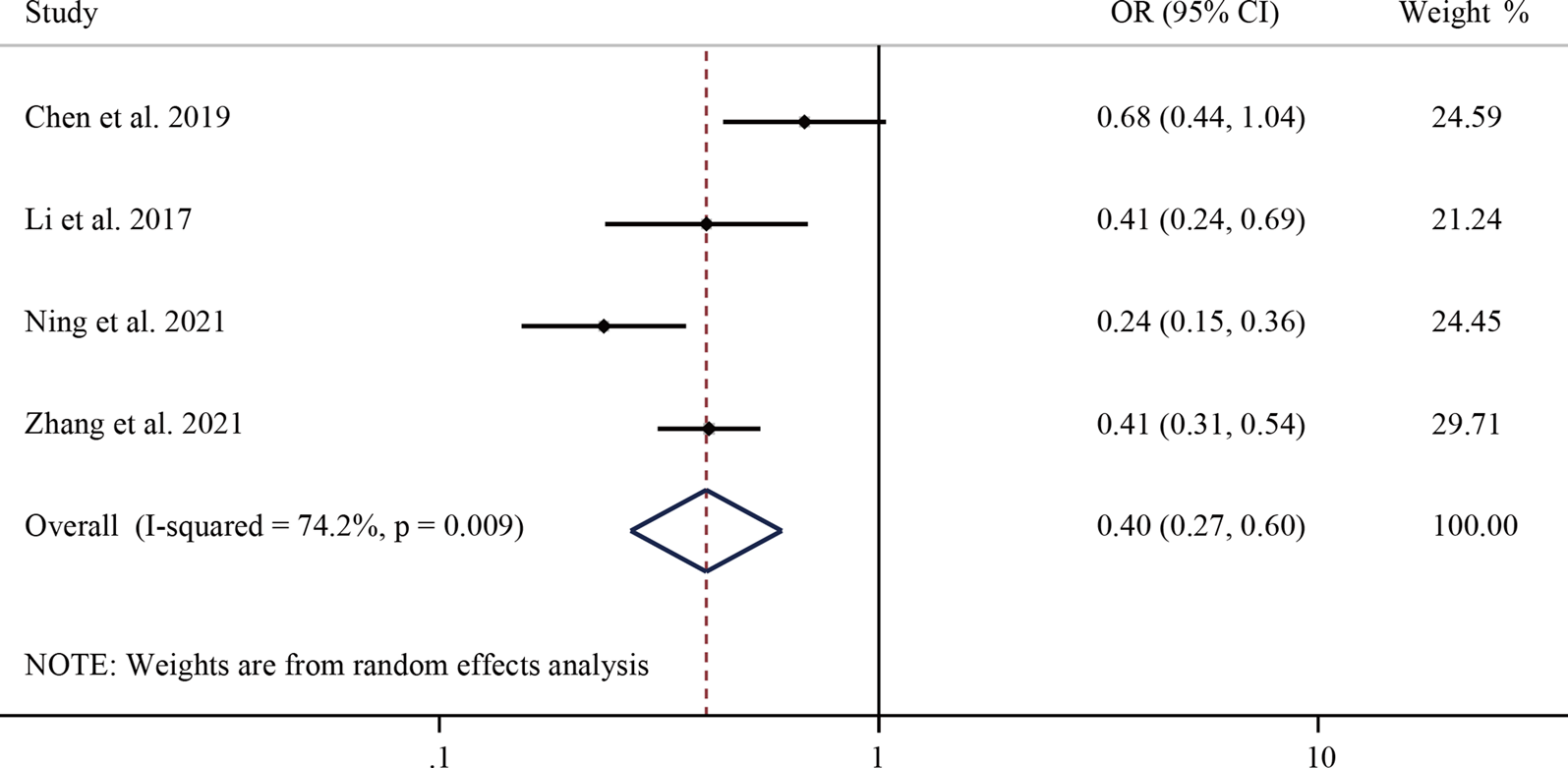
Forest plot of a random-effect model meta-analysis of postoperative anti-osteoporosis therapy as a risk factor for postoperative re-fracture in OVCF patients with PVP/ PKP. OVCF, osteoporotic vertebral compression fracture. PVP, percutaneous vertebroplasty. PKP, percutaneous kyphoplasty

Furthermore, a total of four studies discussed the anti-osteoporosis treatment after PVP/PKP surgery. In Eastern Asia, postoperative anti-osteoporosis treatment was a protective factor for SVCF, as the pooled OR was 0.4 (95% CI 0.27–0.60, *P* < 0.001) (Fig. 6).

### Publication bias

The results of the Begg’s test indicated that no significant publication bias was found in SVCF risk factors, such as gender (*P* = 0.97), age (*P* = 0.23), BMD (*P* = 0.84), and bone cement volume (*P* = 0.37).

## Discussion

The prevalence of osteoporosis increases with age. The total number of osteoporosis is forecast to reach 212 million in China by 2050 [20]. Fracture is the most common and most serious complication of degenerative osteoporosis. Also, OVCF is the most common type of fracture in osteoporosis, even minor trauma may lead to vertebral compression fractures [20]. Osteoporosis has aroused widespread concern due to its serious problems and heavy burden on public health and social economy. With the development of minimally invasive spinal surgery, PVP and PKP have been accepted by many scholars for the rapid response, functional improvement, and better analgesic effect in the treatment of OVCF, and have become increasingly popular in clinical practice [45–47].

Recently, there have been increasing reports of re-fractures after PVP/PKP [48]. It is reported that, in a 36-month follow-up, the incidence of new fractures in patients receiving PVP was 2.2% [13]. A 2-year prospective study indicated that 8 of 42 OVCF patients developed adjacent vertebral fractures after PKP [14]. Moreover, the results of a cohort study suggested that the incidence of re-fractures after PKP was as high as 27.8, and 68% of which occurred in adjacent vertebrae [49]. So far, there are conflicting opinions on whether PVP/PKP increases the incidence of re-fractures in patients. Although many researchers have attempted to explore the relationship between PVP/PKP and postoperative re-fracture through biomechanical studies to determine the risk factors of new vertebral fractures, there are still controversies [44, 50]. Some scholars believe that this is the result of the natural progression of osteoporosis, while others believe that PVP/PKP treatment may increase the risk of re-fracture or degenerative changes in adjacent vertebrae [51–53].

This study is a comprehensive and systematic review of the existing literature. The results of this meta-analysis, including 9144 OVCF patients from 22 studies, show that the incidence of vertebral re-fracture after PVP/PKP is 13.46%. Also, female, advanced age, low bone mineral density, high AP ratio, high Cobb ratio, high VAS and bone cement leakage are risk factors for re-fracture. In addition, postoperative anti-osteoporosis treatment is a protective factor for re-fracture.

Advanced age and low bone mineral density are strong risk factors for re-fracture after PVP/PKP. Chen et al. determine that advanced age and low bone mineral density are risk factors for postoperative re-fracture [33]. A study conducted in Singapore reports that advanced age may increase the risk of re-fracture after percutaneous vertebroplasty [54], as BMD and bone mass decrease with age. Moreover, postoperative anti-osteoporosis treatment can increase bone mineral density, reduce calcium loss, and significantly reduce the probability of postoperative re-fracture.

Female is a risk factor that cannot be ignored for re-fractures. For women, postmenopausal osteoporosis carries a high risk of fractures. Different drugs and administration methods may be more effective than others in preventing certain complications or clinical outcomes, but the best pharmacological treatment options remain unclear. A meta-analysis of randomized controlled trials investigated that Denosumab had the best treatment effect, and Raloxifene and Alendronate had a lower incidence of serious adverse events overall [55]. A previous study has found that PVP could change the biomechanics of the spine, thereby increasing the compression of adjacent vertebral bodies and intervertebral discs, especially in women with severe osteoporosis [56]. Moreover, low BMI is an important risk factor for increased bone loss in postmenopausal women, which further increases the risk of postoperative fractures [57]. Bone turnover markers (BTMs) highlight delicate balance between bone formation and resorption [58–60]. Procollagen type I N propeptide (PINP) is a marker of bone formation, and cross-linked C-telopeptides of type I collagen (bCTx) is considered a marker of bone resorption [59, 61]. It has been found inversely associated between BMD and serum levels of PINP [62]. Greater CTx values may lead to a reduction in BMD and T-score [63, 64]. BTMs are considered to have potential clinical utility as therapy monitors and prediction tools for complications [63, 65].

At present, there is no unified conclusion on the amount of bone cement injected during the operation, but more bone cement is not better. Excessive filling of bone cement may increase the pressure load of the adjacent vertebral bodies, leading to subsequent fractures [66]. Also, the results of a retrospective study have found that patients with less bone cement filling have a higher risk of re-fracture after surgery [34]. Moreover, there is increasing evidence that the amount of bone cement is not associated with the occurrence of new cone compression fractures [42, 67]. The results of our study show similar results.

Bone cement leakage includes intervertebral disk extravasation, paravertebral extravasation and epidural leakage. As a common complication of PVP/PKP, bone cement leakage is considered by most scholars to be related to vertebral re-fracture after surgery. Although it has been reported that the frequency of adjacent vertebral fractures in the bone cement leakage group and the non-leakage group after PVP is roughly the same [68]. However, some studies have supported the view that bone cement leakage is a risk factor for re-fracture of the vertebral body after PVP/PKP surgery. Gao et al. have found that paravertebral and intradiscal subtype of cortical leakage are an important risk factor for new vertebral compression fractures and recompression [69]. This study supports the view that bone cement is a risk factor.

There are some limitations to this meta-analysis. Firstly, the included literatures are retrospective study. This can cause considerable deviations and potentially affect our results. Secondly, the majority of patients included are Asians, which may not be representative of all risk factors. In all included studies, only one patient is from Europe, and the rest are from Asia, concentrated in China, Taiwan, and South Korea. Time effects such as study duration and timing can lead to inaccuracies. Furthermore, some potential risk factors are not included in this analysis due to inaccurate records or insufficient reports, which would have a certain impact on the research results.

## Conclusion

In summary, the results of this meta-analysis suggest that gender, age, bone mineral density, AP ratio, Cobb ratio, postoperative VAS and bone cement leakage are the major risk factors for re-fracture after PVP/PKP, especially in Eastern Asia. Also, anti-osteoporosis treatment is a protective factor. These results may have profound implications for clinical practice and research. Moreover, based on these risk factors, the surgeon could optimize the patient’s preoperative condition and develop a more comprehensive treatment plan. Furthermore, after the operation, regular and detailed follow-up and timely measures should be taken to prevent complications. Obviously, this meta-analysis integrates the results of known risk factors, therefore, future research could focus on undiscovered risk factors.

## Data Availability

The datasets used and/or analysed during the current study are available from the corresponding author on reasonable request.

## Abbreviations

PVP: Percutaneous vertebroplasty
PKP: Percutaneous kyphoplasty
OVCF: Osteoporotic vertebral compression fractures
OR: Odds ratio
WMD: Weighted mean difference
SVCF: Secondary vertebral compression fractures
BMD: Bone mineral density
BMI: Body Mass Index
PRISMA: Preferred reporting items for systematic reviews
MOOSE: Meta-analysis of observational studies in epidemiology
NOS: Newcastle–Ottawa Scale
VCF: Vertebral compression fractures
VAS: Visual Analog Scale
BTM: Bone turnover marker
PINP: Procollagen type I N propeptide
bCTx: Cross-linked C-telopeptides of type I collagen
